# Supplementary figures and images for: Transcriptome association studies of neuropsychiatric traits in African Americans implicate PRMT7 in schizophrenia
Source: PeerJ. 2019 Sep 26;7:e7778. doi: 10.7717/peerj.7778 (PMC6766368; doi:10.7717/peerj.7778)

## Schizophrenia PCA Mapping

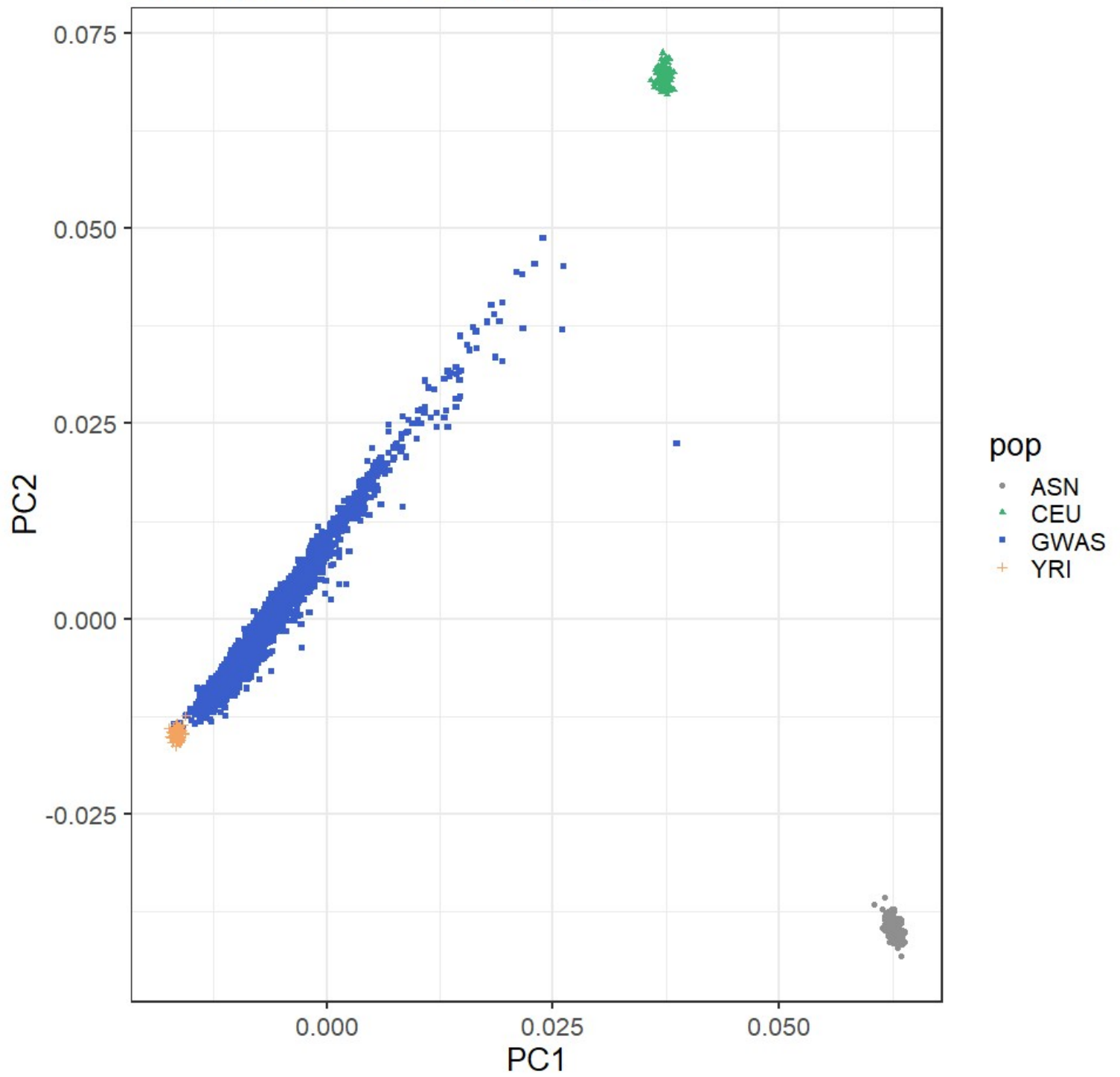

Supplement: Supplemental Information 1 — We performed principal component analysis on the GAIN cohort merged with three populations from version three of the HapMap Project. Each point on the plot represents one individual in the study plotted across axes for their first and second principal components. The three HapMap populations plotted are Chinese in Beijing and Japanese in Tokyo (ASN), European ancestry in Utah (CEU), and Yoruba people in Ibadan, Nigeria (YRI). [file peerj-07-7778-s001.pdf]

Bipolar Disorder PCA Mapping

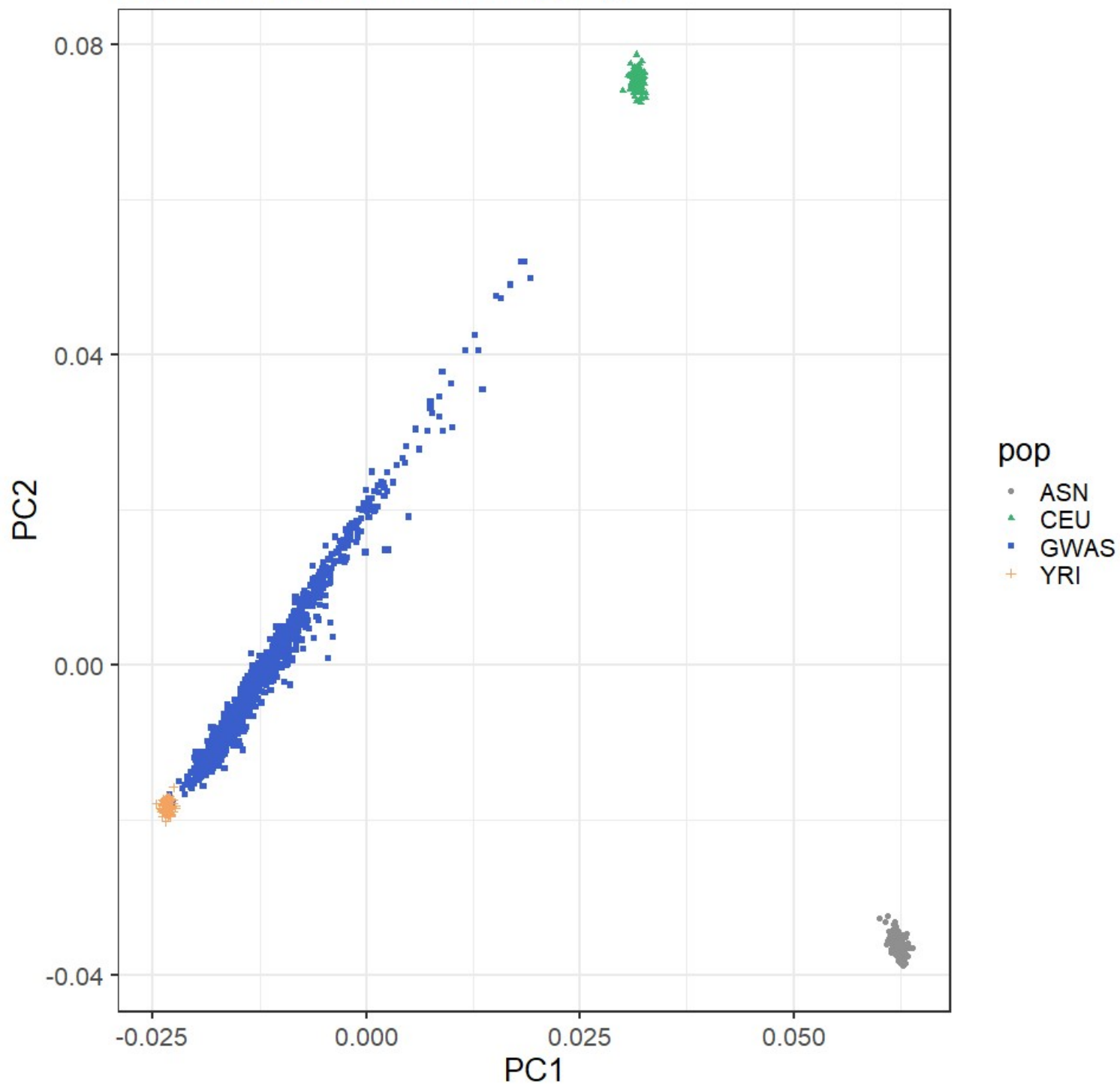

Supplement: Supplemental Information 2 — We performed principal component analysis on the GAIN cohort merged with three populations from version three of the HapMap Project. Each point on the plot represents one individual in the study plotted across axes for their first and second principal components. The three HapMap populations plotted are Chinese in Beijing and Japanese in Tokyo (ASN), European ancestry in Utah (CEU), and Yoruba people in Ibadan, Nigeria (YRI). [file peerj-07-7778-s002.pdf]

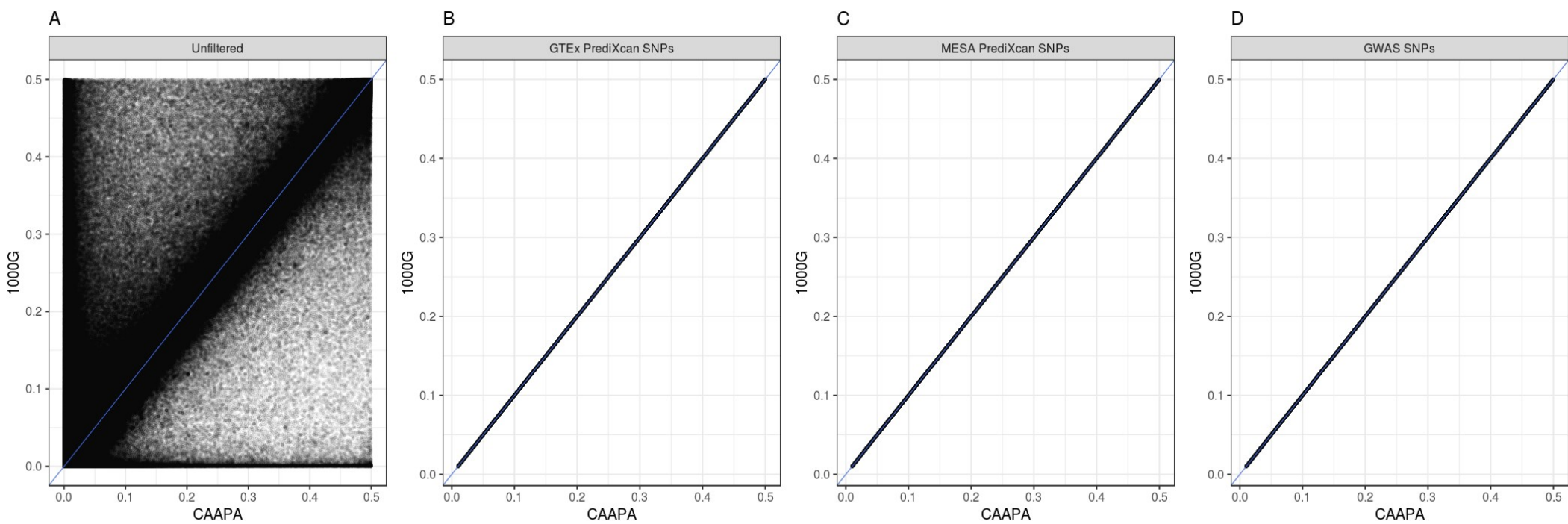

Supplement: Supplemental Information 3 — We imputed genotypes using the University of Michigan Imputation Server using either 1000G or CAAPA as the reference panel. (A-D) depict the MAFs of SNPs from the GAIN schizophrenia study. We saw a similar pattern of MAFs in the GAIN data of the bipolar disorder study. (A) depicts the MAF of SNPs at the intersection of each reference panel before filtering by r2 > 0.8 and MAF > 0.01. (B) Depicts MAFs of SNPs in 1000G and CAAPA from (A) that passed the filters of r2 > 0.8 and MAF > 0.01and were included in the GTEx prediction models across 44 tissues. (C) shows a plot of the MAFs of filtered SNPs from 1000G and CAAPA found in the MESA predictors. (D) shows a plot of the MAFs of filtered SNPs from 1000G and CAAPA that were included in our GWAS. [file peerj-07-7778-s003.pdf]
